# Uso de um método não invasivo no monitoramento da pressão intracraniana em unidade de terapia intensiva para melhorar a neuroproteção em pacientes no pós-operatório de cirurgia cardíaca após circulação extracorpórea

**DOI:** 10.5935/0103-507X.20210066

**Published:** 2021

**Authors:** Salomón Soriano Ordinola Rojas, Amanda Ayako Minemura Ordinola, Viviane Cordeiro Veiga, Januário Manoel de Souza

**Affiliations:** 1 Unidade de Terapia Intensiva Neurológica, BP - A Beneficência Portuguesa de São Paulo - São Paulo (SP), Brasil.

**Keywords:** Procedimentos cirúrgicos cardiovasculares, Lesões encefálicas, Pressão intracraniana, Circulação extracorpórea, Neuroproteção, Cuidados críticos

## Abstract

Desde a instituição da circulação extracorpórea, há cinco décadas, a lesão cerebral decorrente desse procedimento durante cirurgias cardiovasculares tem sido uma complicação frequente. Não existe uma causa única de lesão cerebral pelo uso de circulação extracorpórea, porém se sabe que acomete cerca de 70% dos pacientes submetidos a esse procedimento. A avaliação da pressão intracraniana é um dos métodos que podem orientar os cuidados com os pacientes submetidos a procedimentos associados com distúrbios neurológicos. Este artigo descreve dois casos de pacientes submetidos à cirurgia cardiovascular com circulação extracorpórea, para os quais os procedimentos de neuroproteção na fase pós-operatória foram guiados pelos achados relacionados ao formato das ondas de pressão intracraniana, obtidos por meio de um método não invasivo de monitoramento.

## INTRODUCTION

Extracorporeal circulation (ECC) has been used in cardiac procedures for five decades, and although continuous efforts have been made to improve the technique, its use can cause mild to severe postoperative complications, including death.^(1)^

Neurological postoperative complications (temporary or long-lasting) can be found in 70% of patients evaluated by specialists. These neurological complications range from cognitive impairment to brain death and include ischemic stroke, which is related to high mortality rates (33% die after one year and 53% die within five years).^(2^-^4)^

Regarding preoperative factors, it has been suggested that age may play a role in the development of brain lesions, whereby older individuals present higher risks; nevertheless, the mechanisms of brain lesion development are not completely understood. In addition, other factors can be related to previous diseases associated with the impairment of cerebral blood flow autoregulation (e.g., diabetes), systemic arterial hypertension and chronic renal failure. Such conditions may cause greater oxygen extraction during ECC, leading to brain hypoxia and resulting in permanent neurological damage.^(4)^

Postoperative factors have been suggested to be related to hypoxia and rewarming temperature after the use of ECC; however, the findings in the literature are inconclusive.^(4)^

Intracranial pressure (ICP) assessment is one method that can guide the management of patients undergoing procedures associated with neurological disturbances. However, the invasiveness of the standard methods for ICP monitoring, requiring the placement of sensors directly in contact with the brain tissue, and their associated risks to the patient (such as infections, brain tissue lesions, hemorrhage) contribute to a scenario where ICP is not a widely considered brain parameter, apart from critical conditions such as traumatic brain injury.^(5)^

Several potential methods for the noninvasive assessment of ICP have been described. The different methods vary as to whether they can provide accurate ICP absolute values (in mmHg), the characteristic ICP pulse waveform and the ability for continuous monitoring.^(6)^ In this scenario, specific characteristics of ICP may be assessed by a noninvasive method independent of its capability to yield absolute values, such as the ICP waveform.

This is a case report of two patients who underwent cardiovascular surgery with ECC and in whom clinical neuroprotection protocols were applied in the postoperative phase and guided by ICP waveform findings obtained with a novel noninvasive ICP monitoring device.

As the patients presented here are part of a wider study sample involving noninvasive ICP monitoring in an intensive care unit (ICU) environment, which is registered under protocol number #89534718.0.0000.5483/2018 with the local ethics committee, the patients gave their consent to participate in the wider study and for the publication of the case report. Cardiac surgery was an elective procedure.

### Noninvasive intracranial pressure assessment

**Figure 1:**
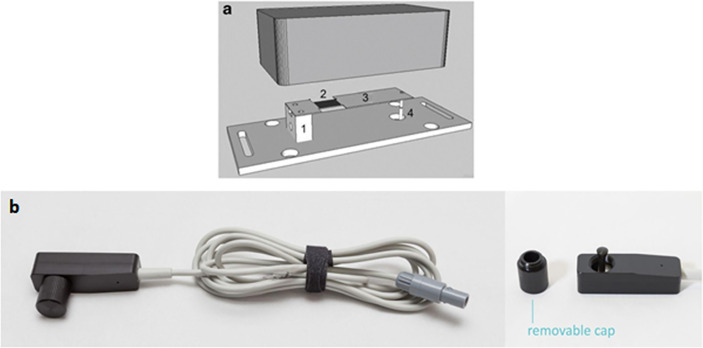
(A) Schematic drawing of all components of the noninvasive system: (1) support for the sensor bar, (2) strain gauge sensor, (3) cantilever bar (sensor bar), and (4) pin. (B) Picture of the noninvasive sensor.

Source: picture courtesy of Brain4care®.

**Figure 2:**
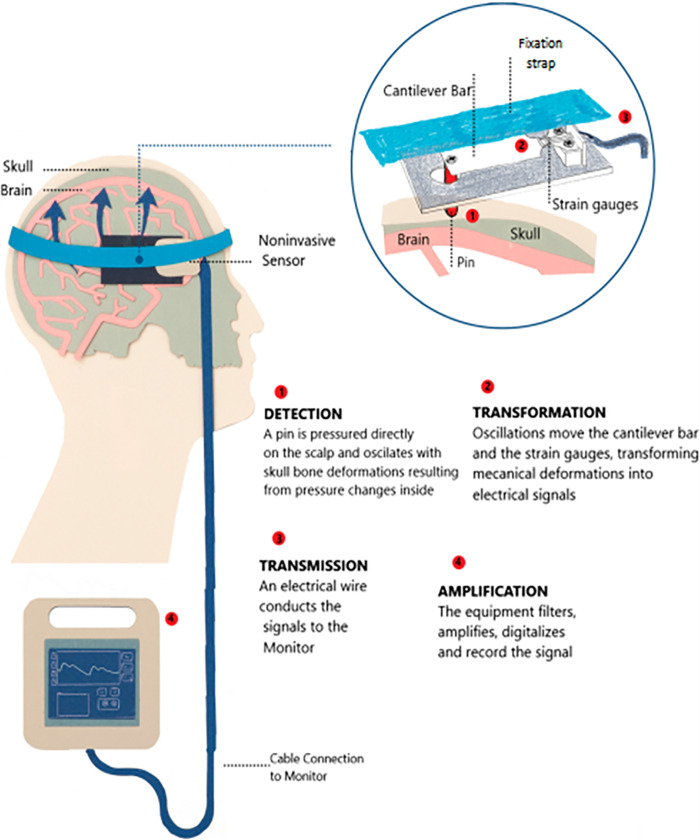
Noninvasive sensor for intracranial pressure monitoring. Signal acquisition steps, schematic details of sensor components, head fixation mode and communication with the multiparameter monitor.

Source: picture courtesy of Brain4care®.

The novel noninvasive method for ICP monitoring (Brain4care®, Brazil) consists of a strain gauge fixed on a mechanical device that touches the surface of the scalp in the frontoparietal region lateral to the sagittal suture (Figure 1). The device can detect slight changes in the dimensions of the skull resulting from ICP changes without the need for surgical procedures. Skull movements are micrometric, and the sensor contains electronic and software tools that are capable of capturing and treating such signals to provide the ICP waveform (Figure 2).

At the current stage of development, this method cannot provide direct ICP absolute values; however, a complete ICP waveform with all the characteristic peaks can be obtained. In addition, the method allows continuous monitoring. The ICP waveform presents with three characteristic peaks: P1 (the percussion wave, due to arterial pressure being transmitted from the choroid plexus to the ventricles), P2 (the tidal wave, related to brain compliance), and P3 (the dicrotic wave, related to the closure of the aortic valve during diastole).^(7)^ Under normal ICP conditions, the amplitudes of these three peaks are obtained as P1 > P2 > P3. Nevertheless, in conditions with decreased brain compliance and rising ICP, the pulse waveform morphology gradually changes, and certain indicators, such as the P2/P1 ratio, eventually increase, denoting a picture of intracranial hypertension (a P2/P1 ratio > 0.8).^(6^-^8)^

This method has been validated in two ways through a direct comparison with invasive methods in animal experimentation,^(6)^ wherein a correlation value of 0.8 was obtained, and through a direct comparison in critical care patients requiring invasive ICP monitoring,^(9)^ which showed a greater similarity between the ICP waveforms obtained with a noninvasive method and those obtained with an invasive methods than between the peripheral arterial blood pressure measurements obtained with a noninvasive method and those obtained with an invasive method. The noninvasive sensor for monitoring ICP was placed over the frontotemporal region of the patient’s head without the need for trichotomy. The signal was collected for at least 5 minutes and a maximum of 30 minutes, with the patient in a supine position with the head of the bed elevated at 30°. The patient’s head was also positioned to secure craniocervical alignment for adequate venous drainage of the jugular veins. The noninvasive ICP signal was sent to the Brain4care® automated analysis system, and after processing, a PDF report containing the P2/P1 ratio results was returned.

## CASE REPORT

### Case 1

A 40-year-old male was diagnosed with Marfan’s syndrome 9 years ago with a previous history of aortic dissection correction. The patient was admitted to the hospital with aortic endocarditis and underwent valved conduit exchange and coronary artery reimplantation.

During the surgical procedure, the patient was maintained in ECC for 182 minutes. The aorta was clamped for 9 minutes 112 minutes after the beginning of surgery. This procedure has been associated with marked decreases in cerebral blood flow and brain hypoxemia.^(1)^

After the procedure was finished, the patient was transferred to the ICU, sedation and mechanical ventilation were maintained, and vasoactive drugs were administered. On the same day, the patient had a single episode of a tonic-clonic seizure, presenting bilateral mydriasis during this episode. After seizure cessation, the pupils remained isocoric, and rapid foot movements (RFMs) were present. Noninvasive ICP monitoring was requested to evaluate the effectiveness of the neuroprotection measures that were used. In the institutional protocol to manage patients with suspected intracranial hypertension, all patients are monitored with invasive blood pressure and ICP monitoring (invasive and/or noninvasive) with the following targets: ICP < 20mmHg, cerebral perfusion pressure - 60 - 70mmHg, sodium > 140mEq/L, partial pressure of carbon dioxide (PaCO_2_) 35 - 40mmHg, temperature < 37.0°C, and glucose 140 - 180mg/dL.

The patient was monitored 4 times, starting just after the seizure episode, and the three subsequent monitoring sessions occurred once a day throughout the following days, according to the institutional protocol.

### Findings and medical management

**Figure 3:**
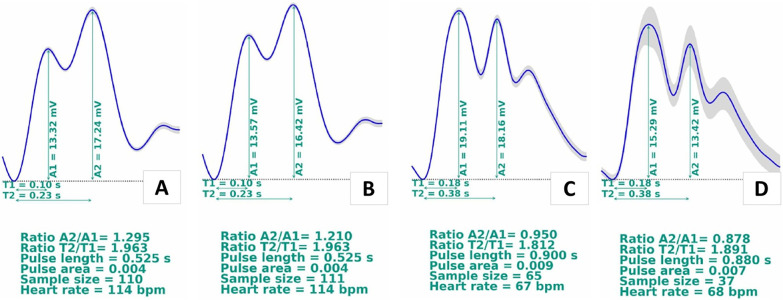
Intracranial pressure waveform monitoring: (A) monitoring after the surgery (day 0); (B) monitoring after 24 hours; (C) monitoring after 48 hours; (D) monitoring after 72 hours.

**Figure 4:**
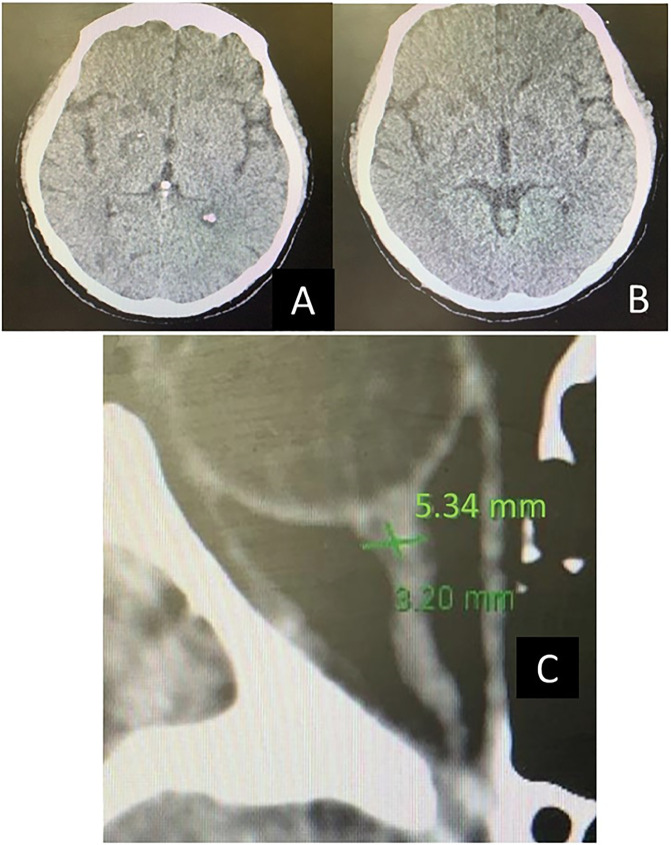
Brain computed tomography: (A) computed tomography scan on the first day; (B) computed tomography scan on day 4, showing an improvement in punctual edemas; (C) optic nerve sheath measurement, with an abnormal diameter of 5.34mm.

**Figure 5:**
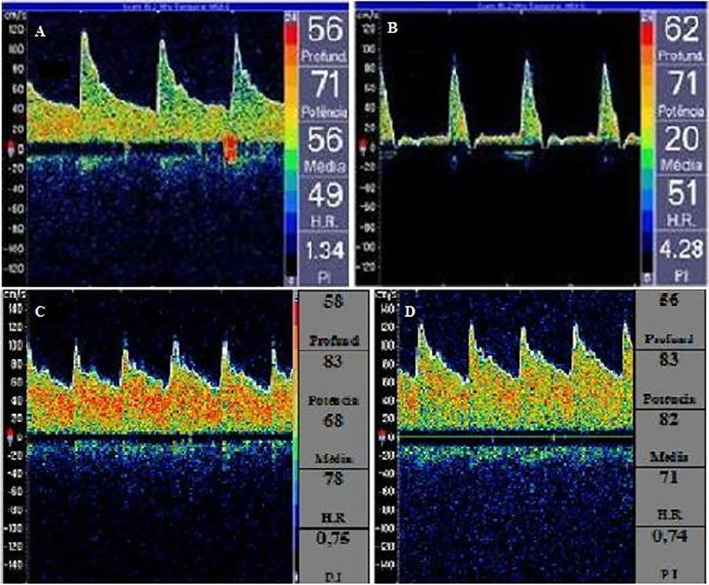
Transcranial Doppler. (A) An increase in cerebral vascular resistance and an increase in the pulsatility index with “spiked waves”. (B) Jugular vein maneuver causing an increase in the resistive index. (C and D) Improved flux waves after treatment.

Immediately after the seizures, the monitoring showed an indication of altered brain compliance, with an averaged waveform showing P2 > P1 (P2/P1 ratio = 1.29, Figure 3A). Brain computed tomography (CT) (Figure 4) showed diffuse cerebral edema and an optic nerve sheath measurement with an abnormal diameter of 5.34mm. The electroencephalogram (EEG) revealed diffuse disorganization at first. Transcranial Doppler assessments of the middle cerebral arteries (MCAs) revealed normal mean cerebral blood flow velocities (98 cm/s and 78 cm/s for the right and left brain hemispheres, respectively), without an indication of microemboli or clear signs of intracranial hypertension but with varying mean cerebral blood flow velocities in the MCAs, which could be related to cerebral autoregulation impairment (Figure 5A). To overcome the potential detrimental implications of dysfunctional autoregulation in cerebral perfusion pressure, we targeted a systolic blood pressure of 140mmHg and a mean arterial blood pressure of 100mmHg.

The medical management included an increase in the sedation medications and the prevention of secondary cerebral ischemia by using vasoactive drugs to keep the systolic blood pressure above 140mmHg.

The second monitoring, 24 hours after the first one showed an indication of maintained altered brain compliance, with P2 > P1, and marginal improvement in the P2/P1 ratio (P2/P1 = 1.21, Figure 3B). Transcranial Doppler assessments of the cerebral arteries revealed abnormal mean cerebral blood flow velocities (Figure 5B).

The medical management included the maintenance of the sedation and vasoactive drug schemes for neuroprotection. Blood pressure targets were established according to transcranial Doppler findings (Figure 5C).

The third monitoring, 24 hours after the second showed improvement of the brain compliance, with a P2/P1 ratio under 1.0 (P2/P1 = 0.95, Figure 3C), still above the threshold for altered brain compliance (P2/P1 > 0.8), with maintenance of the sedation and vasoactive drug schemes. Another noninvasive ICP monitoring was requested after 48 hours.

The fourth monitoring, 48 hours after the third showed that the patient responded well to the neuroprotection management, without any signs of intracranial edema on a tomography exam performed on the same day. The noninvasive ICP waveform appeared almost normalized (P2/P1 = 0.88, Figure 3D), and transcranial Doppler revealed normal mean cerebral blood flow velocities.

The medical management involved weaning from the mechanical ventilation and a sedation medication withdrawal scheme.

The patient was extubated on the fifth day postsurgery and did not present any neurological complications.

### Case 2

A 50-year-old male had a history of aortic aneurysm repair 2 years ago. The patient was admitted to the hospital for the correction of an aortic dissection. During the procedure, he underwent ECC for 82 minutes, with aortic clamping for 6 minutes 48 minutes after the beginning of the surgery.

After the procedure was finished, the patient was transferred to the ICU, sedation and mechanical ventilation were maintained, and vasoactive drugs were administered following the same institutional protocol described previously.

Noninvasive ICP monitoring was requested to evaluate the effectiveness of the neuroprotection measures that were used. The patient was monitored three times: immediately postsurgery, 24 hours postsurgery and 48 hours postsurgery.

### Findings and medical management

**Figure 6:**
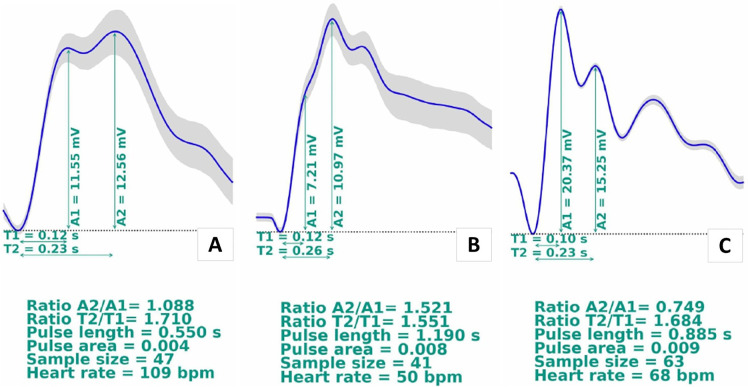
Intracranial pressure waveform monitoring, with an improvement in the intracranial pressure pulse: (A) monitoring after the surgery (day 0); (B) monitoring after 24 hours; (C) monitoring after 48 hours. The results showed an improvement in the intracranial pressure pulse morphology on day 2.

First monitoring, immediately postsurgery: an indication of altered brain compliance, with an averaged waveform showing P2 > P1 (P2/P1 = 1.09, Figure 6A). Brain CT showed diffuse cerebral edema, the initial eye examination revealed miosis progressing to isocoric pupils throughout the entire ICU stay, and the EEG did not reveal any epileptic activity. Transcranial Doppler examination was not requested for this patient.

The medical management included maintenance of the sedation scheme (midazolam associated with fentanyl), according to the institutional protocol for intracranial hypertension and a request for another noninvasive ICP monitoring after 24 hours.

Second monitoring, 24 hours after the first monitoring: an indication of maintained altered brain compliance, with P2 > P1, with a worsening of the P2/P1 ratio (P1/P2 = 1.52, Figure 6B).

The medical management involved a change in the sedation scheme by including dexmedetomidine instead of midazolam associated with fentanyl and a request for another noninvasive ICP monitoring after 24 hours.

The third monitoring, 24 hours after the second showed brain compliance within the normal range (P2/P1 = 0.75, Figure 6C).

Mechanical ventilation weaning process and a sedation withdrawal scheme were started.

The patient was extubated on the third day postsurgery and did not present any neurological complications.

## DISCUSSION

This exploratory study brings new technology to the cardiac ICU environment concerning different aspects of the cardiac postoperative period. These patients were managed using noninvasive methods such as noninvasive ICP monitoring, transcranial Doppler and optic nerve sheath measurement. The potential central nervous system hypoflux (low cerebral blood flow) caused by ECC during cardiac surgery can lead to secondary brain damage,^(1)^ and these noninvasive alternatives could promote early identification of detrimental intracranial dynamic changes, thus enabling appropriate neuroprotective measures to be implemented in postoperative ICU care.

The sedation depth was assessed using the Richmond Agitation Sedation Scale (RASS).^(10)^ Both patients were sedated to a depth of RASS-4/-5 to avoid mechanical ventilation desynchrony.

In case 2, the worsening of the P2/P1 ratio after 24 hours was probably a result of the edema development process.

The use of dexmedetomidine was analyzed and published by Schomer et al. in 2019,^(11)^ who showed the safety and efficacy of the drug as a useful adjunctive agent in the treatment of refractory hypertension. This drug can reduce the use of hyperosmolar boluses, therefore decreasing the risk of intravascular volume derangements, acute kidney injury and rebound intracranial hypertension. For cardiac patients, it is suggested that the use of dexmedetomidine can also promote adequate heart rate control.

## CONCLUSION

It is believed that the reported surgical procedures are associated with intermittent brain hypoxia, which could lead to eventual cerebral edema, increased intracranial volume with consequent derangement of brain compliance and intracranial hypertension due to hypoxic ischemic brain injury. The patients presented in this study did not meet the criteria to receive an invasive intracranial pressure catheter because anticoagulants were administered during cardiac surgery. The noninvasive method for intracranial pressure monitoring identified morphological changes in the intracranial pressure waveform postsurgery and throughout patient management in the intensive care unit. In these two reported cases, this noninvasive method assisted in clinical decision-making regarding the optimization of protocols adapted for neuroprotection.
